# Data-driven identification of metabolic and cardiovascular biomarkers in high-altitude workers: a machine learning approach

**DOI:** 10.3389/fpubh.2025.1652605

**Published:** 2025-11-28

**Authors:** Ricardo Jorquera, Guillermo Droppelmann, Gonzalo Blanco, Max Dollmann, Ignacio Ahumada, Felipe Feijoo

**Affiliations:** 1 Workmed, Santiago, Región Metropolitana, Chile; 2 Clínica MEDS, Santiago, Región Metropolitana, Chile; 3 School of Industrial Engineering, Pontificia Universidad Católica de Valparaíso, Valparaíso, Chile

**Keywords:** artificial intelligence, cardiovascular biomarkers, fitness-for-work, high-altitude, machine learning

## Abstract

**Background:**

Workers in high-altitude mining settings face increased cardiometabolic risk due to chronic exposure to low oxygen levels. Traditional fitness-for-work (FFW) assessments often evaluate biomarkers in isolation, missing relevant health patterns.

**Aim:**

To improve the risk stratification of the FFW status in high-altitude workers by identifying relevant biomarkers through ML models.

**Methods:**

A retrospective cohort of 420,966 preemployment examination records, corresponding to 89,149 workers between 2021 and 2024 was analyzed. Workers were classified as fit or unfit for work, in each of their medical examinations, according to national guidelines. Several supervised ML models were applied, including random forests (RF), support vector machines, k-nearest neighbors, and decision trees, to identify relevant predictors of FFW. Logistic regression was performed to assess statistical associations between biomarkers and fitness outcomes.

**Results:**

Among the 420,966 preemployment examination records, 48,783 were particularly assessed for fitness for high-altitude work. Among these, 8% were classified as unfit for high-altitude work. Significant predictors included body mass index (BMI), blood glucose, triglycerides, and systolic blood pressure. The Random Forest (RF) model outperformed SVM and KNN, achieving the highest predictive performance with an accuracy of 0.89, sensitivity of 0.92, and specificity of 0.83. Multivariate logistic regression confirmed BMI as the strongest predictor (OR 2.640, *p <* 0.001), followed by glucose (OR 2.000, *p* < 0.001), triglycerides (OR 1.461, *p* < 0.001), systolic blood pressure (OR 1.380, *p* < 0.001), smoker (OR 1.125, *p* < 0.002).

**Conclusion:**

ML models can effectively identify critical health indicators related to FFW in high-altitude environments. These tools offer the potential to improve occupational health assessments and support preventive decision making in vulnerable worker populations.

## Introduction

Chile, one of the leading copper producers in the world, employs approximately 993,000 workers in the mining sector, with more than 80% stationed at elevations greater than 3,000 meters above sea level (1, 2). These high-altitude (HA) environments expose workers to chronic hypobaric hypoxia, which triggers acute and long-term physiological responses (3). Due to the limited ability of the human body to adapt, workers are at increased risk of developing chronic mountain sickness, pulmonary hypertension, and cardiovascular disease (CVD) (4).

To protect worker health, Chile has implemented specific regulations, such as Law 16.744 on occupational diseases and Supreme Decree No. 28/2012, which address risks associated with chronic intermittent hypobaria (5, 6). In 2013, the Ministry of Health (MINSAL) published technical guidelines mandating comprehensive medical evaluations before employment in HA settings, with mandatory renewals at fixed intervals (7, 8). These evaluations include clinical and laboratory tests that cover anthropometry, cardiovascular and renal function, and complementary evaluations such as audiometry, ophthalmologic, and psychological tests (9).

However, the current approach to evaluating fitness-for-work (FFW) is fragmented. Variables are often assessed in isolation using predefined cut-off points, which may not capture the complexity of physiological interactions. This reductionist strategy may overlook individuals at risk, and severe incidents, including fatalities, have occurred even under full regulatory compliance. Acute mountain sickness (AMS) remains a concern in HA miners. Although its incidence is markedly lower than in short-term visitors, symptomatic episodes can still occur in workers deemed fit, sometimes after years of employment. Evidence from a nested case-referent study in Kyrgyzstani miners demonstrated that current smoking was a very strong independent risk factor for severe AMS (OR *≈* 10) (10). Integrative screening algorithms, such as those explored in this study, could reduce AMS by better filtering susceptible individuals. Although BMI is not a direct risk factor for AMS in miners, identifying and excluding vulnerable workers by multivariate biomarker analysis can improve safety for both employees and employers.

Given these limitations, there is growing interest in complementary tools that capture complex health dynamics. Artificial intelligence (AI), particularly machine learning (ML), has shown potential in enhancing diagnostic precision, early detection, and risk stratification in multiple clinical areas. In medicine, ML has improved diagnostics of musculoskeletal imaging, prediction of ECG arrhythmias, and risk stratification for chronic diseases (11–13). In occupational medicine, ML applications are still emerging, but promising studies have demonstrated its ability to predict accidents and identify key health risk factors (14, 15).

However, validation of these tools in occupational cohorts of HA remains scarce. Traditional approaches, such as the Framingham Risk Score, although widely used, may not adequately reflect the unique biomarker profiles seen in miners working at altitude (16). Furthermore, limited research has addressed the prediction of long-term health deterioration or loss of FFW in this specific population (17). This highlights the need for data-driven integrative models that synthesize multiple health indicators to provide a more comprehensive perspective of worker health.

In this study, FFW was defined according to the national technical guidelines of the MINSAL and the Social Security Superintendency (SUCESO) for chronic intermittent hypobaric hypoxia (Decree No. 28/2012; MINSAL, 2013). Workers were categorized as fit or unfit for high-altitude employment based on the presence of one or more medical or laboratory contraindications established in these official protocols.

## Materials and methods

### Study design and data extraction

This observational study followed the guidelines of the Strengthening Observational Studies in Epidemiology Reporting Framework (STROBE) (18). A retrospective cohort design with an exploratory scope was used, encompassing all preemployment health assessments of workers exposed to HA environments. These evaluations, conducted by a private health provider operating in multiple regions of Chile, spanned the second half of 2021 to the first half of 2024. According to national legislation, these assessments are mandatory within the first 30 days of employment and must be renewed annually for workers to remain eligible to work at HA.

### Dataset characteristics

**Figure 1:**
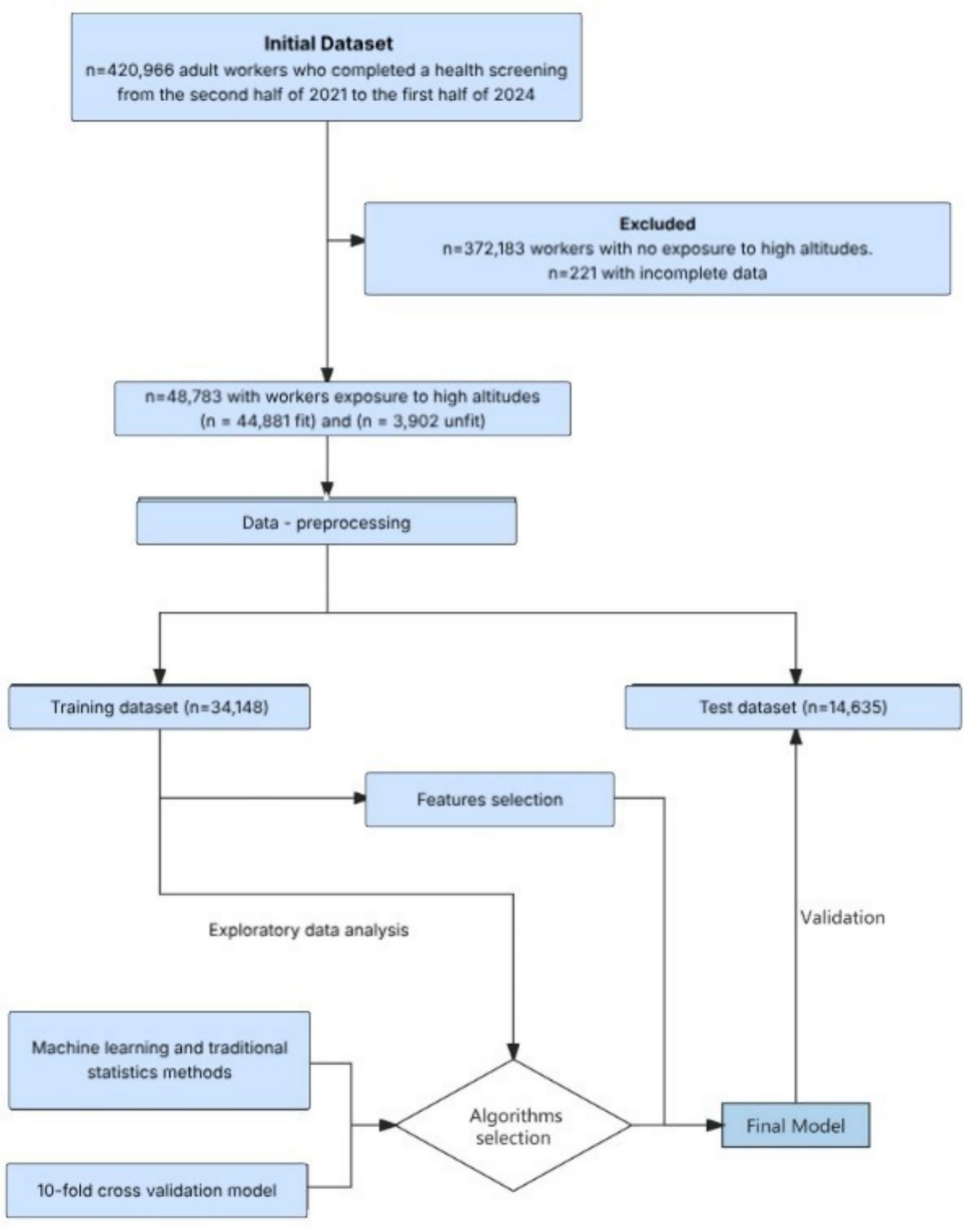
Flowchart of dataset.

An extensive database supplied by a private healthcare provider was analyzed to assess the results of preemployment screening batteries for workers exposed to HA conditions. These conditions include any work activity performed in offices or branches of the employer located 3,000 meters above sea level or higher. Workers were classified according to their health fitness for the HA work as fit (without contraindications) or unfit (with contraindications) according to the guidelines issued by the SUCESO (19) and the MINSAL Department of Occupational Health Technical Guidelines (7). Figure 1 illustrates the data flow used in this study.

### Data preparation

Data cleaning and preparation were performed to ensure the quality and consistency of the information. The data set was processed in R using packages such as *dplyr* and *tidyr* to streamline the workflow (20). Missing values were addressed through imputation methods (median or mode depending on the nature of the variable) and outliers were identified and managed to minimize their impact on the analysis. Duplicate entries were removed and inconsistencies, such as formatting errors or invalid data, were corrected. Categorical biomarkers were converted to a suitable format and continuous biomarkers were standardized to meet the requirements of the selected models (21).

### Definition of fit and unfit criteria

All preemployment examinations of workers exposed to HA were identified in the database. The tests included medical and laboratory evaluations, considering biomarkers such as age, sex, BMI, glucose levels, triglycerides, total cholesterol, HDL cholesterol, heart rate, systolic and diastolic blood pressure, and cardiovascular risk calculated using the Framingham index. Additional parameters, such as hemoglobin and creatinine, were evaluated, along with complementary tests, including electrocardiograms, audiometry, and visual examinations.

Physical activity was assessed by self-report during the structured preemployment health interview. Workers were classified as physically active if they participated in at least three moderate intensity exercise sessions per week, consistent with occupational health recommendations (22, 23). Smoking status was also self-reported. Workers were considered smokers if they reported daily cigarette consumption. Occasionally smokers with less than daily use were also included in this category. Former smokers were classified as non-smokers, according to national occupational health guidelines. The use of waterpipes and electronic cigarettes was not systematically recorded in the data set and therefore could not be analyzed separately. Biochemical verification (e.g., exhaled carbon monoxide) was not performed, as this is not required in Chilean preemployment evaluations (24).

In accordance with the Chilean MINSAL and SUCESO guidelines, preemployment examinations did not include systematic measurement of peripheral oxygen saturation (SpO2) at the first ascent, nor repeated monitoring of SpO2 at each shift. Spirometry was also not performed routinely, restricted to cases with specific respiratory symptoms or previous pulmonary disease. These differences highlight the variations between Chilean protocols and those applied in other high-altitude settings, such as Kyrgyzstan, where both SpO2 and spirometry are mandatory (25, 26).

### Model development and feature selection

#### Random forest and decision trees

To identify critical biomarkers associated with worker fitness for HA environments, RF was applied as one of the primary models. RF is particularly effective in medical data analysis due to its capacity to handle numerous variables simultaneously while minimizing overfitting. The ensemble structure of the model, built through repeated sampling and randomized feature selection, ensures stable predictions even in noisy datasets. One of the added benefits of RF is its internal mechanism for evaluating the relevance of characteristics, which was leveraged to classify biomarkers according to their predictive contribution. Mean Decrease in Accuracy criteria was used to assess variable importance.

To complement RF analysis, a single decision tree (DT) model was developed. Unlike RF, DT offers direct interpretability by producing a hierarchical sequence of decision rules. These rules allowed for the identification of specific cutoff points in continuous variables, such as BMI and systolic blood pressure, which played a role in determining whether a worker was deemed fit or unfit for exposure to HA. This model was particularly useful for visualizing the reasoning behind certain classifications and helped clarify the decision thresholds suggested by the RF model.

#### Support vector machines and k-nearest neighbors

Additional classification models were used to compare performance between different algorithmic approaches. SVM were chosen for their ability to create robust classification boundaries in high-dimensional feature spaces. By maximizing the margin between fit and unfit categories, SVM helps to improve generalizability and resist overfitting, especially when the input space is complex. In parallel, the k nearest neighbors (KNN) algorithm was applied as a simple instance-based classifier. KNN assigns class membership based on proximity in feature space, considering the most common outcome among the nearest observations. Although less sophisticated than other approaches, KNN serves as a useful comparator due to its intuitive nature and ability to capture local patterns.

#### Logistic regression models

To support the classification results and provide interpretable statistical associations, a LR analysis was also performed. This method estimates the probability of an individual being classified as unfit based on a linear combination of selected biomarkers and is valued for its interpretability and broad acceptance in clinical research. Given the number of predictors involved, multicollinearity and overfitting were potential concerns. To mitigate this, univariate LR models were first applied to identify significant predictors. These variables, together with those ranked as important by the RF and DT models, were then included in the multivariate LR analysis. Ultimately, this approach allowed for quantifying the independent contribution of key variables and yielded clinically relevant insights consistent with the results of the ML algorithms. Finally, based on prior evidence, smoking status was included as a covariate in the multivariate logistic models, regardless of its ranking in the machine learning feature importance analysis.

#### Training procedure and hyperparameter tuning

To address data imbalance, defined as a significantly larger number of samples in the majority class (fit) compared to the minority class (unfit), different balancing strategies were implemented, such as oversampling, downsampling, and hybrid approaches. Balancing techniques were also implemented during the K-fold cross-validations and overfitting evaluation. In particular, a 10-fold repeat cross-validation with external data balancing approaches was performed. This approach allowed the model to learn from both classes in an equitable way, improving its overall performance and reducing potential bias (27). The hyperparameters of the model were optimized during the cross-validation process using grid search techniques to improve classification performance and prevent overfitting.

For RF models, number of trees was kept at 1, 50, 100, and 500, while mtry (number of variables selected for branching) was optimized using grid search. Other hyperparameters were kept with their default values. For the case of SVM, hyperparameters were optimized automatically using caret’s internal tuning procedure (tune length = 10), which evaluates combinations of corresponding hyperparameters through cross-validation and selects the configuration yielding the highest classification accuracy. For SVM with linear kernel, the cost parameter (C) was optimized, while for the radial kernel, both C and the kernel width (*σ*) were tuned.

The data set was divided into a training set (70%) and a testing set (30%) to evaluate the model performance. This split ensured that the testing set remained representative of the original dataset while the training set underwent the balancing process. By combining these strategies, we minimized the impact of data imbalance and improved the reliability of the model predictions.

#### Model evaluation and statistical analysis

Statistical analyses and graphical representations were performed using the R statistical software package (version 4.5.0). The caret (version 7.0–1) and randomForest (version 4.7–1.2) packages were used for model training, tuning, and performance evaluation.

A significance level of *p* < 0.05 was applied to determine statistical relevance. The results of the LR models were reported using odds ratios and *p*-values to highlight the strength and significance of the associations.

Descriptive statistical methods were applied, with categorical biomarkers presented as frequencies and percentages, and quantitative biomarkers expressed as mean and standard deviation m ± sd. Data normality was assessed using specific tests. Group comparisons were conducted with the Student’s *t*-test or Mann–Whitney *U* test, depending on the data distribution.

ML models, particularly RF, were used to identify the most relevant biomarkers in preemployment examinations of workers exposed to HA. Relevant biomarkers were selected based on Mean Decrease Accuracy criteria. Models were evaluated based on accuracy, sensitivity, specificity, area under the curve (AUC), positive predictive value, and negative predictive value. The binary classifier outputs either 0 or 1, representing the predicted class label. True positives (TP) refer to correctly predicted positive cases, while false positives (FP) indicate instances where the positive class was incorrectly predicted. False negatives (FN) occur when the model incorrectly predicts the negative class, and true negatives (TN) are correctly predicted negative cases (28). Table 1 presents the confusion matrix, which illustrates the four possible outcome scenarios. The McNemar test was applied to evaluate whether the distribution of misclassified cases (false positives vs. false negatives) differed significantly from what would be expected by chance.

**Table 1:** Confusion matrix form binary classification.

		Predicted	
		0	1
Actual	0	True negative (TN)	False positive (FP)
	1	False negative (FN)	True positive (TP)

TN, true negative; FP, false positive; FN, false negative; TP, true positive.

Sensitivity, specificity, and accuracy were calculated using the following formulas.

Sensitivity = TP/(TP + FN).

Specificity = TN/(TN + FP).

Accuracy = (TP + TN) / (TP + TN + FP + FN).

## Results

### Sociodemographic and health characteristics

Our analysis included 48,783 workers who underwent pre-employment examinations to perform tasks under conditions of exposure to HA. The study covered a 35-month period from the second half of 2021 to the first half of 2024. Of these, 93.8% were male and 90.94% were of Chilean origin. Among them, 3,902 workers (8%) were classified as unfit for HA work due to at least one preemployment examination result that fell outside normal levels.

Table 2 shows the distribution of the health biomarkers evaluated during preemployment examinations, stratified by sex. Statistically significant differences were identified in several parameters. Men had higher mean values of glucose, triglycerides, total cholesterol and blood pressure, both systolic and diastolic, compared to women (*p* < 0.001). In contrast, women exhibited higher levels of HDL cholesterol and heart rate, with significant differences observed compared to men (*p* < 0.001). For complementary examinations, men had a higher prevalence of electrocardiograms and audiometric, while no significant differences were observed in visual test results by sex (*p* = 0.65). In terms of habits and lifestyles, the prevalence of smoking, alcohol consumption, and drug use was significantly higher in men (*p* < 0.001). In addition, a higher proportion of men reported participating in regular physical activity compared to women (*p* < 0.001).

**Table 2:** Comparison of health biomarkers by sex overall group included.

Biomarker	Female (*n* = 3,040)	Male (*n* = 45,743)	Overall (*n* = 48,783)	*p*-value
Age (years)	36.3 ± 9	40.8 ± 10.6	40.52 ± 10.56	<0.001
BMI (kg/m^2^)	27.4 ± 4.55	28.4 ± 3.84	28.34 ± 3.90	0.54
Glucose (mg/dL)	89.1 ± 15.5	94 ± 15	93.69 ± 15.08	<0.001
Triglycerides (mg/dL)	115 ± 65.4	158 ± 110	155.32 ± 108.26	<0.001
Cholesterol (mg/dL)	181 ± 36.9	190 ± 40.2	189.44 ± 40.06	<0.001
HDL (mg/dL)	57.2 ± 16	47.2 ± 12.3	47.82 ± 12.79	<0.001
HR (bpm)	73 ± 10.5	70.3 ± 11.1	70.47 ± 11.08	<0.001
Systolic BP (mmHg)	119 ± 11.6	125 ± 10.4	124.63 ± 10.58	<0.001
Diastolic BP (mmHg)	74.8 ± 9.26	78.9 ± 8.2	78.64 ± 8.33	<0.001
CVR (0–1)	0.011 ± 0.004	0.015 ± 0.008	0.014 ± 0.007	<0.001
Hemoglobin (g/dL)	13.9 ± 2.93	15.9 ± 2.69	15.78 ± 2.75	<0.001
Creatinine (mg/dL)	0.78 ± 2.18	0.99 ± 2.78	0.98 ± 2.75	<0.001
ECG (+)	193 (6.3%)	5,840 (12.7%)	6,033 (12.4%)	<0.001
Audiometry (+)	253 (8.3%)	10,029 (21.9%)	10,282 (21.1%)	<0.001
Visual (+)	248 (8.2%)	3,848 (8.4%)	4,096 (8.4%)	0.65
Exercise (+)	1,554 (51.1%)	31,083 (68%)	32,637 (66.9%)	<0.001
Smoking (+)	1,095 (36%)	18,521 (40.5%)	19,616 (40.2%)	<0.001
Alcohol (+)	1,764 (58%)	30,909 (67.6%)	32,673 (67.0%)	<0.001
Drugs (+)	83 (2.77%)	2,155 (4.69%)	2,238 (4.6%)	<0.001

BMI, body mass index; HDL, high-density lipoprotein cholesterol; BP, blood pressure; CVR, cardiovascular risk; ECG, electrocardiogram; HR, heart rate; m, mean; sd, standard deviation; bpm, beats per minute.

### Machine learning models: RF, SVM, and KNN

Several ML models were used to discriminate fit from unfit HA workers. The models were trained on 70% of the data (tested on the remaining 30%), following data balancing techniques and 10-fold cross-validation (see details in the section Methods). Missing data was minimal (0.5%) and was handled based on median or mode imputation. Models were trained with imputed data and with the subset with missing data records removed. Results were indistinguishable due to the minimal amount of missing data. Among the architectures evaluated, the RF model was the architecture that achieved the best performance. The results of the test data set for RF (100 trees) show that the general accuracy was 89% (95%CI: 0.87–0.90) without indication of overfitting. Sensitivity, which reflects the true positive rate, is 92%, while specificity, or the true negative rate, is 83%. The positive predictive value (PPV) is 88.44%, and the negative predictive value (NPV) is 94%. McNemar’s test yielded a statistically significant result (*p* = 1.50 × 10–4), suggesting a difference between misclassified observations. These results suggest that the model is highly effective in distinguishing between the predicted classes, with statistically significant performance metrics (*p* < 0.001). As mentioned above, other models were also evaluated. The SVM model using a radial kernel showed improved performance compared to the linear kernel, with an accuracy of 86%, sensitivity of 91%, and specificity of 81%. In contrast, the linear kernel yielded lower values across all metrics. The KNN model demonstrated good sensitivity (83%) but lower specificity (67%), suggesting that while it captured most unfit cases, it tended to overclassify fit individuals. These findings indicate that while both models contributed to the classification task, their performance was slightly inferior to that of the RF model. The comparison of results for all ML models is shown in Table 3. All of the above models were also trained on a subset of data with not repeated workers. This means that, for workers with more than one medical examinations, only the first medical records were kept. This was done to assess possible influence of workers with repeated examinations on the classification power of the models. Results were consistent, with accuracy, sensitivity, specificity, and AUC on the test set belonging to the confidence intervals shown in Table 3. This guarantees that models generalize well and are not influenced by workers examinations over time (as our unit of analysis is the medical examination, not the worker).

**Table 3:** Performance metrics for machine learning models.

Model	Accuracy	Sensitivity	Specificity	AUC
DT [ntree = 1]	0.83 [0.82, 0.85]	0.82 [0.80, 0.84]	0.85 [0.82, 0.86]	0.82 [0.80, 0.84]
RF [ntree = 50]	0.88 [0.87, 0.90]	0.92 [0.91, 0.94]	0.83 [0.81, 0.86]	0.92 [0.91, 0.94]
RF [ntree = 100]	0.89 [0.87, 0.90]	0.92 [0.91, 0.94]	0.83 [0.81, 0.86]	0.93 [0.92, 0.94]
RF [ntree = 500]	0.88 [0.87, 0.90]	0.93 [0.92, 0.95]	0.83 [0.82, 0.86]	0.93 [0.92, 0.94]
SVM [linear]	0.77 [0.76, 0.79]	0.81 [0.77, 0.82]	0.77 [0.75, 0.80]	0.84 [0.82, 0.86]
SVM [radial]	0.86 [0.84, 0.88]	0.91 [0.88, 0.92]	0.81 [0.80, 0.85]	0.91 [0.90, 0.94]
KNN	0.72 [0.69, 0.73]	0.83 [0.80, 0.86]	0.67 [0.65, 0.70]	0.76 [0.74, 0.78]

RF, random forest; SVM, support vector machine; KNN, k-nearest neighbors.

**Figure 2:**
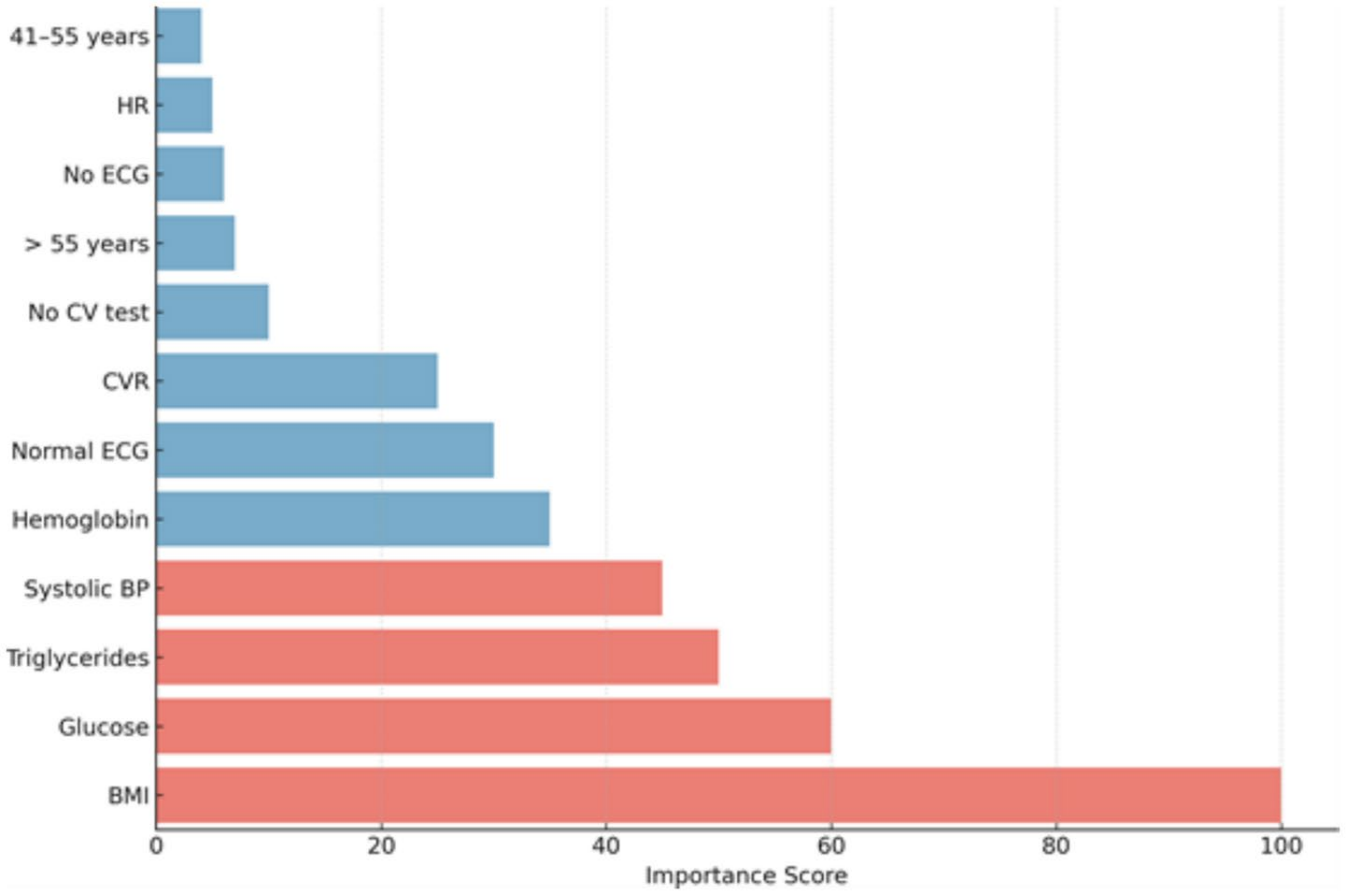
Biomarker importance plot in predictive model for health outcomes. This figure shows the importance of each biomarker in the predictive model. The *x*-axis represents the importance score, ranging from 0 to 100, while the *y*-axis lists the biomarkers used in the model. The biomarkers are ranked based on how much they contribute to the model’s predictive accuracy.

The RF model was also used to identify key biomarkers (as it was the model with the best performance). The RF model identified BMI as the most influential biomarker for fitness evaluation in HA work. Other key biomarkers include blood glucose, triglycerides, and systolic blood pressure. Hemoglobin and a normal electrocardiogram are less important but still showed relatively high importance. Other biomarkers, such as those related to the age of the workers, were of minimal importance. This ranking highlights that metabolic and cardiovascular factors, such as BMI and blood glucose, are the strongest predictors in the RF model. For more details, refer to Figure 2.

### Decision tree classification insights

**Figure 3:**
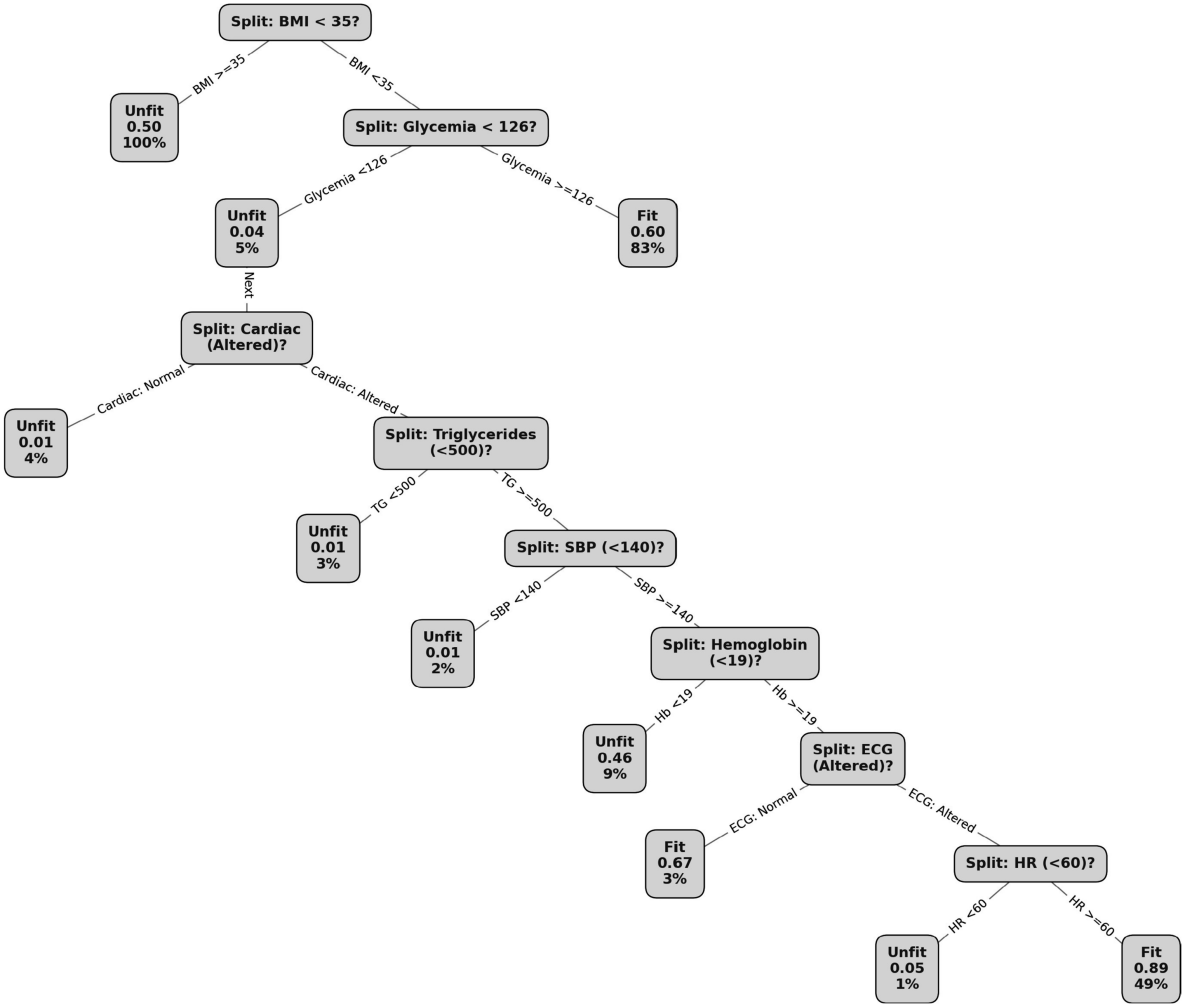
Decision tree from the random forest analysis showing key biomarkers that determine classification into the” Fit” or” Unfit” categories. The model splits first on BMI (≥35) followed by glucose levels (≥126), cardiovascular assessments, triglyceride levels (≥500), systolic blood pressure (≥140), hemoglobin levels, and ECG results. The hierarchical structure highlights the role of metabolic, cardiovascular, and hematologic factors in determining risk.

The DT model based on the RF model was also assessed to understand the relationship between key biomarkers. The DT is illustrated in Figure 3. The figure demonstrates the key biomarkers that contribute to the classification into fit or unfit categories. The initial split occurs according to BMI (≥35), where individuals with a BMI of 35 or higher are classified as unfit. The DT identifies a share of 17% of unfit workers with a BMI greater than 35. For individuals with a BMI below 35, glucose levels (≥126) become the next decision point, followed by further subdivisions based on cardiovascular assessments, triglyceride levels (≥500), and systolic blood pressure (≥140). Additional factors, such as hemoglobin levels and ECG results, refine the classification, particularly for those identified as *fit* to work. The model captures interactions between these biomarkers, progressively narrowing decision paths. The probability of being classified as” Fit” increases as more favorable conditions are met. This hierarchical structure underscores the model approach to risk stratification, focusing on the critical role of metabolic, cardiovascular, and hematologic parameters.

### Logistic regression analysis

The odds ratios derived from the LR analyses offer detailed information on the relationship between individual biomarkers and the probability of being classified as unfit for HA work. In the univariate models, BMI showed a significant association, with each increase in a unit corresponding to a 27 percent increase in the odds of being unfit (OR 1.27). Similarly, blood glucose was associated with a 5 percent increase in the odds per unit (OR 1.05). Triglycerides had a smaller but significant effect (OR 1.01), while systolic blood pressure showed a similar pattern (OR 1.05). These results suggest that, when considered individually, these metabolic and cardiovascular markers contribute modestly but consistently to the risk of unfit to work. Hemoglobin, on the other hand, did not demonstrate a statistically significant association when analyzed alone (OR 1.0, p: 0.919).

The OR for workers (OR 1.00, p: 0.935) who declared that they smoke (or occasionally smoke) was not statistically significant in the univariate model. However, when controlling for this condition, the multivariate model clearly shows a strong association for workers who are considered smokers. Smoke was added to the logistic regression analysis as a controlled variable given its strong evidence in existing research. Similarly, males do not appear to have a higher risk compared to females (OR 0.93, p: 0.339) as shown in the univariate model.

In the multivariate model, where all biomarkers were analyzed simultaneously using standardized values, the strength of the association increased substantially. BMI remained the most influential variable, with a 163 percent increase in the odds of being unfit per increase in standard deviation (OR 2.64, *p*: < 2 × 10^−16^). Blood glucose and triglycerides also showed reinforced effects, with OR of 2.01 and 1.46, respectively (*p*-value for both was < 2 × 10^−16^). Systolic blood pressure maintained its predictive relevance (OR 1.38, *p*: < 2 × 10^−16^). In particular, hemoglobin, which had not reached significance in the univariate model, exhibited a protective association in the multivariate analysis, with a 15 percent reduction in the odds of being unfit (OR 0.85, *p*: 1.63 × 10^−7^). Finally, smoke (positive) shows an increase in the odds of being unfit for work compared to those workers who do not smoke (OR 1.13, *p*: 0.003), while males showed lower risk compared to females (OR 0.54, *p*: 6.58 × 10^−12^) in the multivariate models. For detailed results, see Table 4.

**Table 4:** Odds ratios from univariate and multivariate logistic regression models.

Biomarker	Univariate model		Multivariate model (scaled, 95% CI)	
	OR [95% CI]	*p*-value	OR [95% CI]	*p*-value
BMI	1.299 [1.286–1.313]	<2 × 10^−16^	2.640 [2.527–2.753]	<2 × 10^−16^
Glucose	1.054 [1.051–1.057]	<2 × 10^−16^	2.009 [1.928–2.095]	<2 × 10^−16^
Triglycerides	1.004 [1.004–1.005]	<2 × 10^−16^	1.461 [1.412–1.511]	<2 × 10^−16^
Systolic BP	1.052 [1.048–1.056]	<2 × 10^−16^	1.380 [1.323–1.439]	<2 × 10^−16^
Hemoglobin	1.000 [0.984–1.010]	0.970	0.855 [0.775–0.942]	1.63 × 10^−7^
Smoker (+)	1.000 [0.929–1.069]	0.935	1.125 [1.039–1.217]	0.0033
Sex (male)	0.934 [0.815–1.076]	0.339	0.536 [0.450–0.642]	6.58 × 10^−12^

OR, odds ratio; BMI, body mass index; BP, blood pressure.

The differential behavior of hemoglobin and smoking in univariate and multivariate analyses provides additional information on their physiological roles in high-altitude adaptation. The protective association of hemoglobin, which emerged only after multivariate adjustment, probably reflects the control of confounders such as BMI, blood pressure, and glucose levels. In the univariate context, these variables can obscure the independent contribution of hemoglobin, which under balanced metabolic conditions could indicate an adequate erythropoietic response and a better oxygen transport capacity under chronic hypoxia. In contrast, smoking became significant only after adjustment, suggesting that its adverse effects were initially masked by other variables, particularly BMI and triglycerides. Once these confounders were controlled, smoking revealed its independent contribution to reduced FFW consistent with evidence linking tobacco use to impaired oxygenation and lower acclimatization capacity in high-altitude workers.

## Discussion

Consistent with previous literature, our study identified key factors that influence the health of workers exposed to HA conditions in Chile. ML techniques allowed a detailed exploration of biomarker interactions and critical thresholds derived from preemployment evaluations, especially in cases where individuals were classified as unfit for work. The results underscore the importance of evaluating metabolic and cardiovascular markers collectively, as the multivariate approach not only enhances predictive accuracy but also reveals meaningful relationships that may remain undetected in univariate analyses. This integrative perspective supports more precise screening strategies and the development of targeted interventions to improve both health outcomes and job performance in vulnerable worker populations.

Although this study focuses primarily on HA mining in Chile, the findings have broader implications for other sectors exposed to extreme conditions, such as transportation and construction, where metabolic and cardiovascular risks are also prevalent (29). A systematic review published in 2020 highlighted the increased risk of cardiovascular disease in mining populations exposed to intermittent hypoxia, driven by a combination of socioeconomic and lifestyle factors (30). Although these variables were included in our models, they were not rated highly influential. Instead, algorithms consistently identified BMI as the most impactful biomarker, reinforcing the established role of obesity as a major cardiovascular risk factor in high-risk workers.

Some authors have proposed that BMI plays a pivotal role in adaptation to HA environments (26). Our analysis supports this association, with BMI, blood glucose, triglycerides, and systolic blood pressure emerging as key predictors of FFW under HA conditions. These findings align with previous Chilean studies that emphasize the role of metabolic syndrome and hypertension in acclimatization and neurocognitive function (31).

Among the classification algorithms, the RF model outperformed the others in terms of accuracy and interpretability. DT visualization revealed critical thresholds that help define worker fitness under HA conditions. LR supported these findings by identifying statistically significant associations between BMI, glucose, and blood pressure and the risk of adverse health outcomes. These convergent results validate the reliability of ML models for occupational health screening and reinforce the utility of combining multiple approaches to guide prevention.

The decision tree model identified several thresholds that may appear extreme at first glance, such as triglyceride levels ≥ 500 mg/dL. These cutoff points reflect the model’s data-driven optimization rather than predefined clinical criteria. In the dataset analyzed, this threshold corresponded to a small subgroup of workers with markedly elevated triglycerides who were consistently classified as unfit according to national occupational health guidelines. The presence of such high values emphasizes the nonlinear relationship between metabolic dysregulation and reduced FFW in high-altitude environments. Rather than representing a population norm, these thresholds capture inflection points in risk probability derived from the hierarchical structure of the model. This reinforces the capacity of decision tree algorithms to identify clinically relevant but otherwise underrecognized patterns in heterogeneous occupational health data.

The inclusion of SVM and KNN further strengthened the robustness of the classification framework. Although these models demonstrated slightly lower accuracy than RF, their performance remained consistent. SVM with a radial kernel achieved high sensitivity and specificity. Evaluating different algorithms reinforces the reliability of predictions and facilitates the translation of model outputs into meaningful clinical decisions.

Logistic regression added complementary value by quantifying the independent effect of each biomarker. The consistency between these results and those derived from ML supports the robust ness of our selected predictors and provides clinically meaningful OR, which is valuable to health professionals and decision makers. In particular, multivariate models identified stronger associations than univariate models, suggesting that some biomarkers may be confounded when evaluated in isolation. The concurrent elevation of BMI, glucose, and triglycerides, each linked to increased odds ratios, highlights a synergistic effect that amplifies overall health risk. These findings could inform occupational health guidelines by encouraging risk-based screening tailored to individual biomarker profiles, rather than relying solely on standardized thresholds.

The methodological rigor of this study, including repeated cross-validation, data balancing, and hyperparameter tuning, mitigated overfitting and supported the generalizability of our findings. This comprehensive validation approach improves confidence in the predictive utility of the model and its potential integration into real-world occupational health protocols.

The heterogeneity of the health challenges faced by high-altitude workers highlights the need for more frequent and personalized health interventions, which should guide public health policy decisions. Although current national guidelines, such as those of MINSAL, recommend annual evaluations for workers exposed to chronic intermittent hypobaric hypoxia (19), our findings suggest that this interval may be insufficient to detect early signs of health deterioration. More frequent assessments could improve early detection and lead to more effective preventive strategies in this vulnerable population.

For example, implementing continuous monitoring programs with at least biannual evaluations, complemented by telemedicine technologies, could offer more accessible and responsive care (28). Future research should prioritize longitudinal studies to track health trajectories and assess the long-term effectiveness of such interventions. Furthermore, exploring emerging biomarkers, such as C-reactive protein, salivary cortisol, and HbA1c, may improve the predictive power of existing models by providing insights into systemic inflammation and metabolic stress. In particular, HbA1c, a reliable indicator of long-term glycemic control in individuals with diabetes, could be highly relevant in occupational health settings marked by metabolic dysregulation. Chronic hypobaric hypoxia can exacerbate these imbalances, further increasing susceptibility to adverse outcomes. In addition, integrating wearable health technologies can enhance real-time monitoring and support dynamic, individualized health management strategies (32, 33).

The integration of ML tools into occupational medicine is progressing steadily, sparking interest in their potential to support health monitoring at both the individual and organizational levels. However, the number of scientific studies in this field remains limited. A recent systematic review identified only 15 articles that examined ML applications in occupational health, underscoring the need for continued research (15). National-level studies have already demonstrated the added value of these tools in similar worker populations, especially in contexts where traditional analyzes did not detect significant patterns (34).

In interpreting these findings, it is important to note that the present analysis was based exclusively on internal validation. Although the models demonstrated high performance and robustness within the available dataset, external validation is required in independent cohorts before drawing generalized conclusions or implementing policy changes. Therefore, the recommendations proposed in this study, such as biannual health assessments, potential regulatory updates, and the exploration of wearable monitoring tools, should be regarded as forward-looking perspectives that warrant further investigation. Future studies integrating multicenter data and prospective follow-up designs will be essential to confirm the reproducibility and applicability of these findings in broader occupational settings.

One of the main strengths of this study is its large sample size of nearly 50,000 workers, which enhances the external validity of our results. Furthermore, the use of multiple ML models enabled a comprehensive evaluation of complex biomarker interactions that are difficult to capture using conventional methods (35).

However, certain limitations must be acknowledged. First, retrospective design can introduce selection bias, as data was obtained from a single private healthcare provider. Second, potential confounders such as genetic predisposition, occupational role, or previous acclimatization training were not considered. Furthermore, the cross-sectional nature of the study prevents causal inference, which should be addressed in future prospective research (36).

This study also addressed the potential concern of data leakage arising from workers with repeated medical examinations. To evaluate this, all models were retrained on a subset of the dataset containing only unique workers, where only the first examination per individual was retained. The results of this complementary analysis were consistent with those obtained using the full dataset, with accuracy, sensitivity, specificity, and AUC values remaining within the confidence intervals reported in Table 3. These findings confirm that the classification performance was not influenced by repeated measurements and that the models generalized appropriately, supporting the robustness of the analytical framework and the validity of the conclusions.

Furthermore, the dataset showed a severe skewness towards men, who represented nearly 94% of the study population. This imbalance is consistent with the demographic composition of the Chilean mining sector and other high-altitude occupational settings. Although the large sample size ensured adequate statistical power, the underrepresentation of women limits the ability to draw sex-specific conclusions and highlights the need for future studies that include a larger number of women workers.

A further limitation is the absence of routine measurements of SpO2 in the first ascent and spirometry in the dataset, as these are not required by Chile’s occupational health regulations. Although these parameters are required in other high-altitude occupational contexts, such as Kyrgyzstan (25, 26), their inclusion in future protocols could improve the early detection of hypoxemia and respiratory impairment in Chilean miners.

Finally, another limitation of our study is that lifestyle variables such as smoking and physical activity were self-reported, without objective verification. In particular, smoking status relied on daily cigarette use and occasional smoking, but did not capture waterpipe, or e-cigarette use. Former smokers were grouped with nonsmokers, which may underestimate cumulative tobacco exposure. While these definitions are consistent with Chilean health practice (37), more detailed and standardized assessments, including biochemical validation, could improve the accuracy of lifestyle data in future studies.

## Conclusion

In summary, this study demonstrates the potential of ML to transform occupational health by identifying health risks among workers exposed to extreme environmental conditions such as HA. Through the integration of multiple supervised learning algorithms, we found critical biomarkers, particularly those related to metabolic and cardiovascular function, that are central to fitness classification. The findings of our RF model may serve as a basis for developing more efficient and personalized monitoring systems, supporting the enforcement of existing regulations, and enabling timely evidence-based interventions.

The adoption of advanced tools, such as wearable technologies and emerging biomarkers, offers promising avenues for more continuous and customized occupational health strategies. These innovations may reduce the incidence of HA-related diseases, improve quality of life, and improve regulatory compliance. Ultimately, incorporating data-driven frameworks into occupational health systems could increase worker safety, operational efficiency, and long-term health outcomes in high-risk labor populations.

## Data Availability

The raw data supporting the conclusions of this article will be made available by the authors, without undue reservation.
